# Chemogenetic activation of the HPC-mPFC pathway improves cognitive dysfunction in lipopolysaccharide -induced brain injury

**DOI:** 10.7150/thno.82889

**Published:** 2023-05-11

**Authors:** Chenglong Ge, Wei Chen, Lina Zhang, Yuhang Ai, Yu Zou, Qianyi Peng

**Affiliations:** 1 Department of Critical Care Medicine, Xiangya Hospital, Central South University, Changsha, Hunan Province, China, 410008.; 2 National Clinical Research Center for Geriatric Disorders, Changsha, Hunan Province, China, 410008.; 3 Hunan Provincial Clinical Research Center for Critical Care Medicine, Changsha, Hunan Province, China, 410008.; 4 Department of Anesthesia, Xiangya Hospital, Central South University, Changsha, Hunan Province, China, 410008.

**Keywords:** Cognitive dysfunction, Hippocampus, Medial prefrontal cortex, Sepsis-associated encephalopathy, Lipopolysaccharide

## Abstract

**Rationale:** Although sepsis-associated encephalopathy (SAE) is a common psychiatric complication in septic patients, the underlying mechanisms remain unclear. Here, we explored the role of the hippocampus (HPC) - medial prefrontal cortex (mPFC) pathway in cognitive dysfunction in lipopolysaccharide-induced brain injury.

**Methods:** Lipopolysaccharide (LPS, 5 mg/kg, intraperitoneal) was used to induce an animal model of SAE. We first identified neural projections from the HPC to the mPFC via a retrograde tracer and virus expression. The activation viruses (pAAV-CaMKIIα-hM3Dq-mCherry) were injected to assess the effects of specific activation of mPFC excitatory neurons on cognitive tasks and anxiety-related behaviors in the presence of clozapine-N-oxide (CNO). Activation of the HPC-mPFC pathway was evaluated via immunofluorescence staining of c-Fos-positive neurons in mPFC. Western blotting was performed to determine protein levels of synapse- associated factors.

**Results:** We successfully identified a structural HPC-mPFC connection in C57BL/6 mice. LPS-induced sepsis induces cognitive impairment and anxiety-like behaviors. Chemogenetic activation of the HPC-mPFC pathway improved LPS-induced cognitive dysfunction but not anxiety-like behavior. Inhibition of glutamate receptors abolished the effects of HPC-mPFC activation and blocked activation of the HPC-mPFC pathway. The glutamate receptor-mediated CaMKII/CREB/BDNF/TrKB signaling pathway influenced the role of the HPC-mPFC pathway in sepsis-induced cognitive dysfunction.

**Conclusions:** HPC-mPFC pathway plays an important role in cognitive dysfunction in lipopolysaccharide-induced brain injury. Specifically, the glutamate receptor-mediated downstream signaling appears to be an important molecular mechanism linking the HPC-mPFC pathway with cognitive dysfunction in SAE.

## Introduction

Sepsis-associated encephalopathy (SAE) is a diffuse form of sepsis-induced cerebral dysfunction that occurs in the absence of direct central nervous system (CNS) infection, structural abnormalities, or other types of encephalopathy 1. Patients with SAE exhibit varying degrees of cognitive dysfunction, memory loss, and abnormal behavior, etc 2. However, the underlying pathogenetic mechanisms of cognitive dysfunction in sepsis-induced brain injury have not been well established, and few effective clinical treatments are available. Therefore, elucidating the pathogenesis of sepsis-induced brain injury is an important objective in the search for novel therapeutic targets.

Cognitive function is supported by the coordinated activation of various brain regions, including the hippocampus (HPC) 3, parietal cortices 4, temporal cortices 5, and prefrontal cortex (PFC) 3, 5, 6. The PFC, particularly the medial prefrontal cortex (mPFC), plays a vital role in social cognition and socio-emotional processing 7, 8. Studies using human brain imaging and animal neurophysiological recording have suggested that neural activity in the HPC and mPFC is critical for cognitive tasks 9-12. Numerous studies have reported a direct HPC-mPFC connection, confined to a unidirectional projection from CA1 13 and the ventral two-thirds of the HPC (vHPC) 14. The HPC-mPFC pathway has been implicated in spatial encoding as a component of working memory 3, 15, and has also been associated with Alzheimer's disease (AD) 16, 17, depression 18, 19, and neuropathic pain 20. Working memory defects are a common manifestation of sepsis-induced brain injury. Previous studies have shown that hippocampal dysfunction 21, 22 and prefrontal cortex injury 2, 22 are associated with cognitive dysfunction in sepsis patients. However, to the best of our knowledge, no reports have examined the role of the HPC-mPFC pathway in cognitive dysfunction in sepsis-induced brain injury.

The chemogenetic technology is widely used in remote manipulation of neuronal activity in mice. Designer receptors exclusively activated by designer drugs (DREADD) could be activated specifically by the “designer drug” clozapine N-oxide (CNO). DREADD—namely, mutated human muscarinic receptors (hM3Dq and hM4Di), are engineered G protein coupled receptors (GPCR) which can precisely regulate GPCR signaling pathways 23.

Based on the above-mentioned studies, we hypothesized that the working memory defects in sepsis patients might be associated with dysfunction of the HPC-mPFC pathway, and that activation of this pathway could improve cognitive function in individuals with sepsis-induced brain injury. Previous studies have shown that alpha-amino-3-hydroxy-5-methyl-4-isoxazolepropionic acid receptor (AMPAR) and N-methyl-D-aspartate receptor (NMDAR) subtypes of glutamate receptors were strongly implicated in hippocampus-dependent spatial learning and memory processes 24, 25. Further, glutamate neurotransmission has been found to play an important role in the HPC-mPFC pathway, as well as in recognition memory 26. Accordingly, to determine whether activity of the HPC-mPFC pathway is specifically correlated with that of the postsynaptic glutamate receptor signaling pathway (GRS), and to examine the role of the GRS in SAE, we evaluated the expression of glutamate receptors (AMPAR and NMDAR) and downstream activity of the calmodulin-dependent kinase II (CaMKII)/ cAMP-response element binding protein (CREB)/ brain-derived neurotrophic factor (BDNF)/ tyrosine receptor kinase B (TrKB) signaling pathway.

## Materials and Methods

### Animals

Adult male C57BL/6 mice (initial weight 18-22 g, 6-8 weeks old) were purchased from Hunan SJA Laboratory Animal Co., Ltd. (Changsha, China). Mice were housed in groups of up to five per cage with libitum access to food and water on a 12/12 h light/dark cycle. All animal experiments were approved by the Animal Care and Use Committee of Central South University (No.2019sydw0240). Mice were handled for 2 min per day for 7 days before the behavioral studies took place. Lipopolysaccharide (LPS, 5 mg/kg, intraperitoneal) was used to induce an animal model of SAE.

### Drugs and virus

We used the following compounds in the current study: a lipopolysaccharide (LPS; E. coli 055: B5, L2880; Sigma, USA), Fluoro-Gold (Fluorochrome, LLC, USA), clozapine-N-oxide (CNO; MedChemExpress, USA), D-AP5 (NMDAR competitive antagonist; MedChemExpress, USA), and NBQX (AMDAR inhibitor; MedChemExpress, USA). The doses were as follows: LPS (5 mg/kg dissolved in 0.9% saline, administered via intraperitoneal (i.p.) injection) 27, Fluoro-Gold (4% in 0.9% saline, 0.5 µl/side, administered via microinjection into the mPFC) 28, CNO (1 mM dissolved in 10% DMSO in 0.9% saline, 0.5 µl/side, via microinjection into the mPFC)^29^, D-AP5 (1µg/µl dissolved in 10% DMSO in 0.9% saline, 0.5 µl /side, via microinjection into the mPFC) 30, and NBQX (10 nmol/ml dissolved in 10% DMSO in 0.9% saline, 1 µl /side, via microinjection into the mPFC) 31, according to previous studies.

To activate the HPC-mPFC projection pathway, we bilaterally injected an adeno-associated virus (AAV) that expressed hM3Dq, which is an excitatory designer receptor exclusively activated by CNO. The viruses (pAAV-CaMKIIα-hM3Dq-mCherry and pAAV-CaMKIIα-mCherry) used in this study were verified and purchased from OBiO Technology (Shanghai, China) Corp., Ltd.

### Experimental design

**Figure 1:**
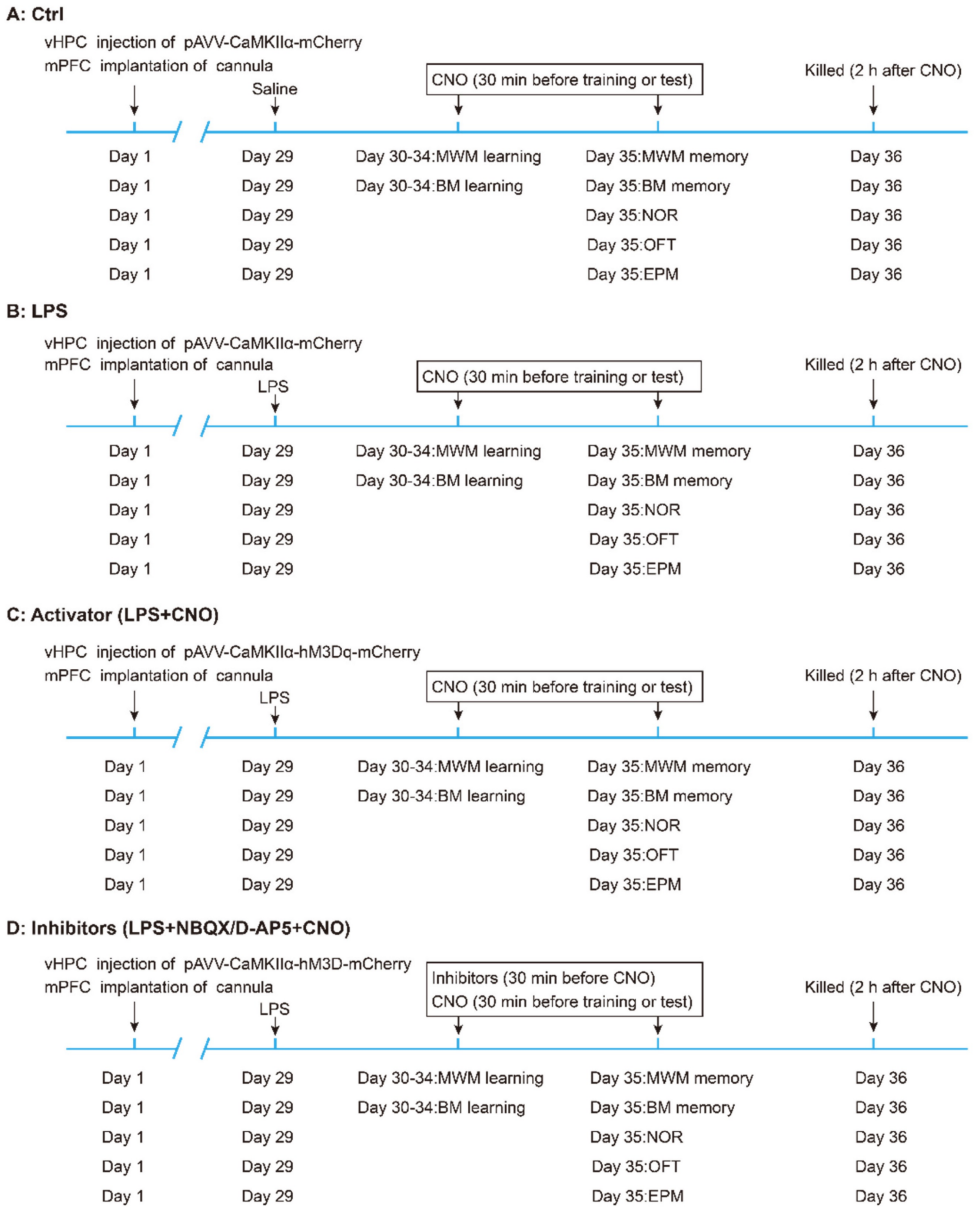
** Flow chart of experimental design.** Mice were randomly divided into five groups: (A) the control (Ctrl) group (n = 30, 6 mice for each behavioral test). (B) the LPS group (n = 30, 6 mice for each behavioral test). (C) the LPS+CNO (Activator) group (n = 30, 6 mice for each behavioral test). (D) LPS+NBQX/D-AP5+CNO (Inhibitors) groups (two sub-groups, n=30/group; with 6 mice for each behavioral test). vHPC, ventral hippocampus; mPFC, medial prefrontal cortex; CNO, clozapine-N-oxide; MWM, Morris water maze; BM, Barnes maze; NOR New object recognition; OFT Open field test; EPM, Elevated plus maze.

The experimental design of the study is summarized in Figure 1. Mice were randomly divided into five groups: (A) the control (Ctrl) group (n = 30, 6 mice for each behavioral test). (B) the LPS group (n = 30, 6 mice for each behavioral test). (C) the LPS+CNO (Activator) group (n = 30, 6 mice for each behavioral test). (D) LPS+ NBQX/D-AP5+CNO (Inhibitors) groups (two sub-groups, n=30/group, with 6 mice for each behavioral test). The sepsis model was induced via an i.p. LPS injection at a dose of 5 mg/kg 27, 32.

### Retrograde tracing

**Figure 2:**
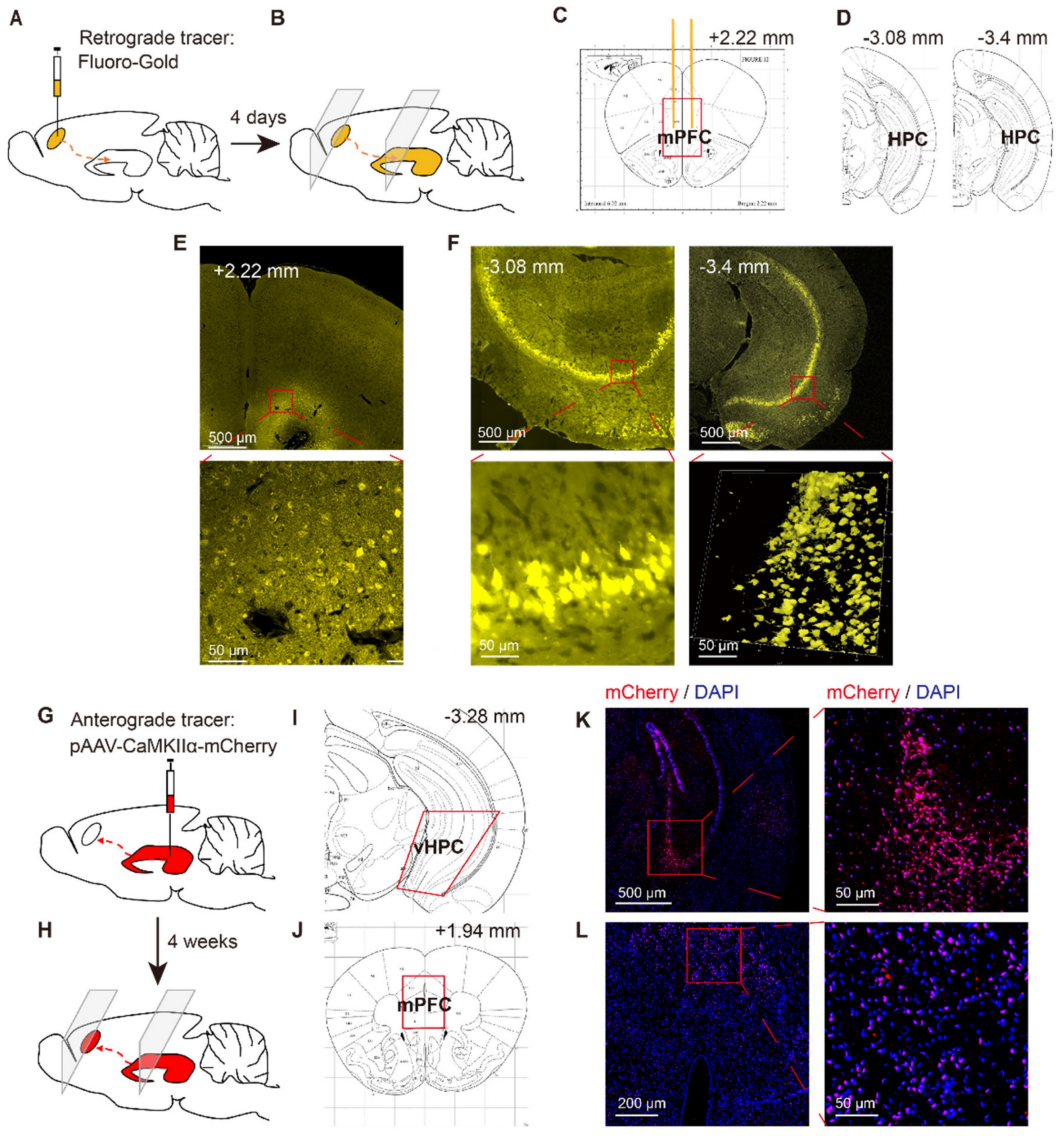
** Identification of HPC-mPFC structural connection in C57BL/6 mice.** (A-F) Retrograde tracing: (A) Retrograde fluorescent tracer (FluoroGold) was injected into medial prefrontal cortex (mPFC); (B) after 4 days, the FluoroGold was mainly shown in ventral hippocampus (vHPC) by coronal sections; (C, D) Representative figures showing the location of the mPFC and vHPC; (E) Representative images showing the FluoroGold in the mPFC; (F) FluoroGold microinjected into the mPFC labeled cells in the vHPC. (G-L) Anterograde tracing: (G) Microinjection of pAAV-CaMKIIα-mCherry into the vHPC; (H) after 4 weeks, the mCherry was mainly shown in ventral mPFC by coronal sections; (I, J) Representative figures showing the location of the mPFC and vHPC; (K) A representative image showing expression of mCherry in the vHPC; (L) Four weeks after viral microinjection into the vHPC, mCherry was detected in the mPFC.

To verify the structure of neurons projecting to the mPFC, another 6 mice were anesthetized via sodium pentobarbital (60 mg/kg, i.p.) and placed in a stereotaxic frame, where they received bilateral injections of the retrograde tracer Fluoro-Gold (4% in saline, 0.5 µl/side) into the mPFC (1.8 mm anterior, 0.4 mm lateral, 1.4 mm ventral) 3 via microinjection needles (33 G). About 4 days after this injection, the mice were anaesthetized via isoflurane inhalation and killed by decapitation. The brain tissue was removed and frozen, and the frozen brain sections (10 µm) were prepared for tracing via Fluoro-Gold in the mPFC and vHPC (3.1 mm posterior, 3.0 mm lateral, 3.9 mm ventral) 3 (Figure 2A~F).

### Stereotaxic microinjection and cannula implantation

Mice were deeply anesthetized with sodium pentobarbital (60 mg/kg, i.p.) and placed into a stereotactic device (RWD Life Sciences, Shenzhen, China). We bilaterally injected 0.5 µl pAAV-CaMKIIα-mCherry (negative control) or pAAV-CaMKIIα-hM3Dq-mCherry into the vHPC (3.1 mm posterior, 3.0 mm lateral, 3.9 mm ventral; 0.5 µl/side) using Hamilton syringes with 33 G needles (Hamilton Reno, NV, USA). To enhance virus expression in the vHPC, we microinjected AAV into a pair of adjacent coordinates for the vHPC (anteroposterior (AP) -3.30, mediolateral (ML) ± 3.30, dorsoventral (DV) -3.60) 33. The viruses were injected using a 33 G needle at a rate of 0.1 µl/min, and the needles were left in place for an additional 5 min to allow for sufficient diffusion.

Bilateral cannulas (OD 0.48 mm, ID 0.34 mm) were implanted 1 mm above the mPFC (AP 1.80, ML ±0.40, DV -1.40) 3. The cannulas were secured to the skull using two stainless-steel screws and dental cement, and 33 G internal cannulas were inserted into the bilateral cannulas to prevent infection and clogging. About 4 weeks after the AVV injection, CNO (0.5 µl/side) or inhibitors were infused bilaterally into the cannulas for more than 1 min using a microinjection needle (33 G) that extended 1 mm beyond the tip of the cannula prior to each trial (including 5 training sessions and 1 testing session). The needles were left in place for over 1 min to allow for adequate drug diffusion.

### Behavioral tests

Behavioral tests began 24 h after the LPS infusion. All behavioral experiments were conducted between 09:00 and 17:00, and each animal was only used in one experimental group.

### Morris water maze

The Morris water maze (MWM) consisted of a circular plastic tank (1.2 m in diameter) filled with water (19-22 °C) and a platform (10 cm in diameter) submerged 1 cm below the surface of the water, as described previously 34. A white food color additive was added to the water to make it opaque and to obscure the platform. The MWM tank was divided into four equal quadrants: northeast (NE), southeast (SE), southwest (SW), and northwest (NW) (Figure 4A). There were visual cues in the room for the mice to use to determine what quadrant they were in. A video camera was positioned above the center of the tank to record the mouse movement traces. We used the MWM to assess the spatial learning and memory abilities of the mice. For the spatial acquisition task, mice were trained over five consecutive days (four trials per day with a 30-s inter-trial interval). CNO (0.5 µl/side) was injected 30 min before trails. In inhibitors group, NBQX (1 µl/side) and D-AP5 (0.5 µl/side) were administrated 30 min before a CNO injection. The mice were gently placed into the water starting at one of four different quadrants (the same quadrant was used in each subsequent trial) and allowed 1 min to find the hidden platform. Each mouse was guided to the platform if it failed to locate the position after 60 s. After the last training session, a probe trial was performed to evaluate spatial memory. In this trial, the platform was removed, and the mice were placed in the water at the same point as in the acquisition trials and allowed to explore freely for 120 s. The escape latency during spatial acquisition, primary latency to the target site, number of platform crossings, time spent in the target quadrant, and swimming speeds and paths in the probe trial were recorded and analyzed using Smart 3.0 software (Figure 4).

### Barnes maze

Barnes maze (BM) consisted of a circular table (92 cm diameter, 105 cm high) with 20 holes (19 empty holes and 1 target hole with escape box) located around the perimeter. As a reference to reach the target hole, we used different cues around the room (black square, yellow triangle, red diamond, and green circle). To prompt mice to complete BM test, bright lights were hung above the center of the maze as negative reinforcement. CNO (0.5 µl/side) was injected 30 min before trails. In inhibitors group, NBQX (1 µl/side) and D-AP5 (0.5 µl/side) were administrated 30 min before a CNO injection. The mice were placed in a cylindrical container at the center of the table for 10 s before each trial. During the learning phase, the mice were allowed to freely explore the target hole for 180 s per trial (3 trials per day) for 5 consecutive days. If a mouse was unable to find the target hole, it was guided towards that location and kept in the escape box for 60 s. After each trial, 75% alcohol was used to wipe and clean the maze to reduce the influence of residual odors on the subsequent test. On day 6, the escape box was removed, and the mice were allowed to freely search the maze for 120 s. During the learning trials, the latencies to the target hole were recorded. During the memory trial, the primary latency to the escape hole, percentage of correct pokes, and total distance moved were recorded and analyzed (Figure 5A~F).

### New object recognition (NOR)

We used a new object recognition (NOR) task to assess recognition memory in the subject mice, as described previously 35, 36. Mice were placed in an empty box (50 × 50 × 50 cm) for 10 min to enable habituation 24 h before testing. Each mouse completed 5 trials (2 minutes each). In the first four trials, the mice were placed inside the box where they were able to explore two identical objects. To reduce odor cues, the arenas and objects were carefully cleaned with 70% alcohol between the trials. On the fifth trial, mice received an injection of CNO (0.5 µl/side) 30 min before the NOR test. In inhibitors group, NBQX (1 µl/side) and D-AP5 (0.5 µl/side) were administrated 30 min before a CNO injection. For the NOR test, one of objects was replaced by a new object. The percentage of time spent exploring the new was calculated (Figure 5G~H).

### Elevated plus maze test

The elevated plus maze (EPM) is commonly used to assess anxiety-related behavior. Here, we conducted the EPM in accordance with a previously described protocol 37. The EPM in this study had two open arms and two enclosed arms, connected by a central platform (5 × 5 cm). Thirty min after a CNO injection, mice were placed on the central platform facing an open arm and allowed to freely explore for 5 min. In inhibitors group, NBQX (1 µl/side) and D-AP5 (0.5 µl/side) were administrated 30 min before a CNO injection. The number of entries to the arms and the time spent on the arms were separately measured (Figure 6A~E).

### Open field test

At 30 min post-CNO injection, mice were subjected to an open field test (OFT) as a way to estimate anxiety-related behavior. In inhibitors group, NBQX (1 µl/side) and D-AP5 (0.5 µl/side) were administrated 30 min before a CNO injection. The mice were gently placed in the corner of a plastic chamber (40 × 40 × 40 cm) and allowed to move freely for 5 min. The time spent in the center area (25 × 25 cm) and total distance traveled in this region were recorded (Figure 6F~H).

### Immunofluorescent staining and image analysis

Immunofluorescent staining was performed as described in a previous study 29. At 120 min after a microinjection of CNO, mice were anesthetized with 1% sodium pentobarbital (100 mg/kg, i.p.) and perfused with 0.9 % saline followed by 4 % paraformaldehyde. In inhibitors group, NBQX (1 µl/side) and D-AP5 (0.5 µl/side) were administrated 30 min before a CNO injection. Subsequently, the brains were rapidly removed and fixed overnight in 4% paraformaldehyde, and then sectioned coronally with a sliding microtome into frozen sections (10 µm thick) at the vHPC and mPFC level. For the c-Fos experiment, the tissue sections were washed 3 × 10 min in phosphate-buffered saline (PBS) followed by a 50 min incubation period with blocking buffer (3% BSA in PBS, 0.1% Triton X100). The sections were then incubated at 4°C overnight with the following primary antibodies, diluted using a commercial antibody diluent (NCM Biotech, China): anti-c-Fos (1:500, ab208942, Abcam, USA) and anti-mCherry (1:500, ab183628, Abcam, USA). On the following day, the sections were washed 3 times with PBS and incubated with the corresponding secondary antibodies diluted in PBS (1:500) for 60 min. The secondary antibodies included Alexa Fluor 488 (ab150113, Abcam, USA) and Alexa Fluor 594 (ab150080, Abcam, USA). After washing 3 times with PBS, sections were counterstained with 4′,6-Diamidino-2-phenylindole (DAPI) and observed using a fluorescence microscope. Images were captured using a confocal microscope. The number of c-Fos positive cells was counted using Image J software. For the Fluoro-Gold experiment, tissue sections were directly observed and photographed using ultraviolet light (380 nm excitation wavelength, 420 nm absorption wavelength) under a fluorescence confocal microscope.

### Western blotting

The brain regions containing the HPC and mPFC were carefully isolated using ophthalmic micro-tweezers and stored at -80 °C until use. The proteins were separated via SDS polyacrylamide gel electrophoresis (SDS-PAGE) and probed with appropriate antibodies against NR2A (1:500), NR2B (1:500), NR1 (1:1000), TrKB (1:1000), GluA1 (1:1000), GluA2 (1:1000), CaMKII (1:1000), CREB (1:1000), pCREB (1:500), BDNF (1:1000), and GAPDH (1:5000). All of these antibodies were purchased from Bioworld Technology (Bioworld, Louis Park, MN, USA). Blotting was detected with secondary antibodies and visualized via chemiluminescence using an enhanced chemiluminescence (Omni-ECL) detection reagents kit (Epizyme Biotechnology, Shanghai, China). The protein bands were quantitatively analyzed using Image J software.

### Statistical analyses

Data were presented as mean ± standard error of the mean and analyzed using GraphPad Prism 9.0 software (GraphPad software, San Diego, CA, USA). Behavioral tests, c-Fos results, and WB values were analyzed using one-way analysis of variance (one-way ANOVA). Time series data were analyzed with repeated measures two-way ANOVA. Following a D'Agostino-Pearson's test, determining the normality of the distributions, multiple comparison between the groups was performed using post hoc Tukey-Kramer method. P < 0.05 was considered statistically significant.

## Results

### Identification of a structural HPC-mPFC connection in C57BL/6 mice

To verify the neural projections from the HPC to mPFC, a retrograde fluorescent tracer (Fluoro-Gold) was microinjected into the mPFC of C57BL/6 mice (Figure 2A, C, E). After 4 days, the Fluoro-Gold tracing was predominantly visible in the HPC area, as viewed in serial coronal sections (Figure 2B, D, F). To further examine direct monosynaptic inputs from the HPC to the mPFC via anterograde tracing, we microinjected pAVV-CaMKIIα-mCherry into the vHPC and visualized the neural terminals in the mPFC (Figure 2G, I, K). After 4 weeks, we observed extensive expression of mCherry (red) in the mPFC (Figure 2H, J, l).

### Chemogenetic activation of the HPC-mPFC pathway

**Figure 3:**
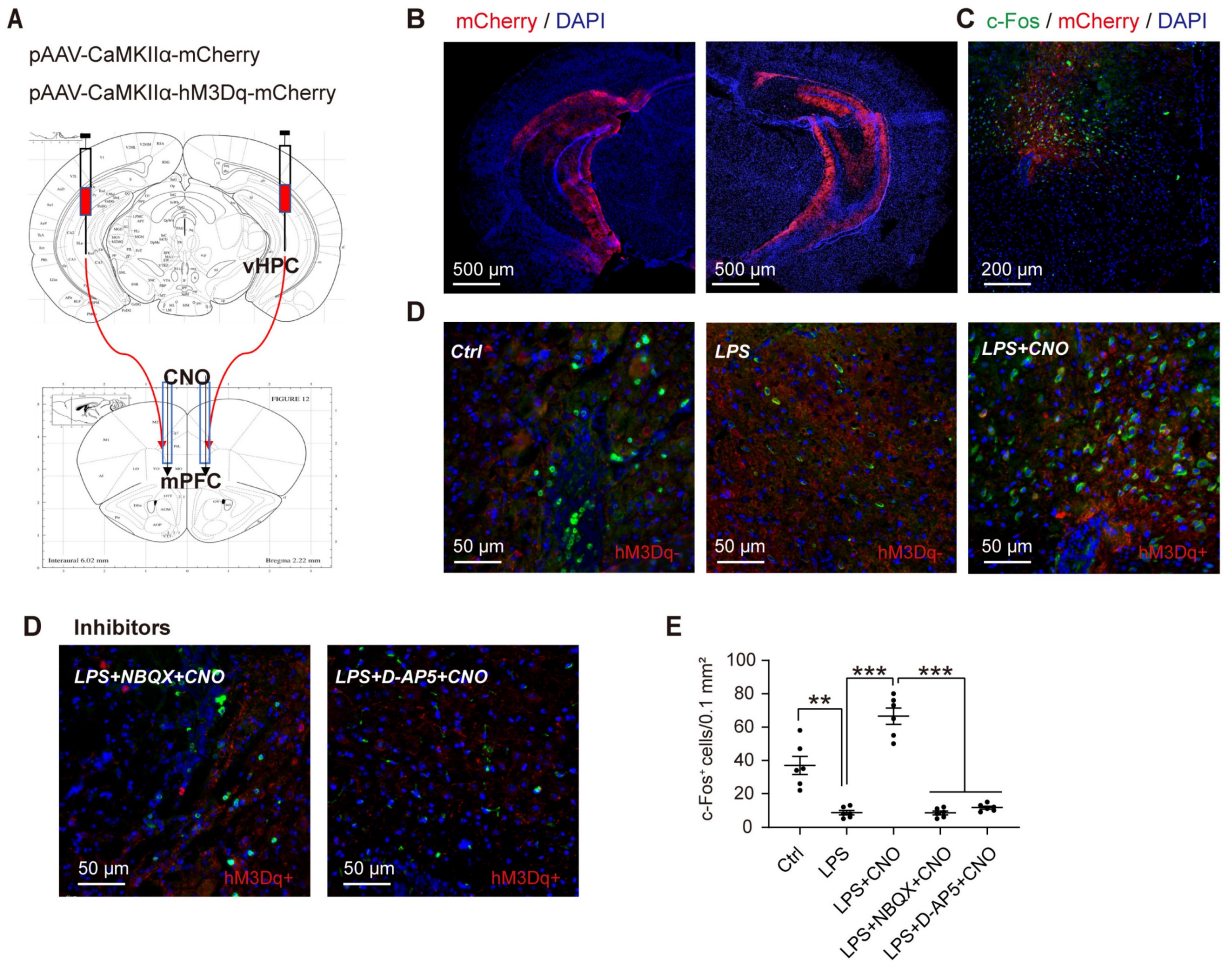
** Activation of the HPC-mPFC pathway.** (A) Representative figure showing chemogenetic activation of the HPC-mPFC pathway. (B) Representative images showing expression of hM3Dq-mCherry and -mCherry in the HPC. (C) Representative image showing c-Fos and mCherry expression in the mPFC. (D) Representative images showing c-Fos expression in the mPFC of different groups. (E) Chemogenetic activation of the HPC-mPFC pathway increased the number of c-Fos-positive cells in the mPFC of septic mice (LPS+CNO vs. LPS, *** P<0.001); Compared with the Ctrl group, the expression of c-Fos is significantly lower in LPS group; Compared with the LPS+CNO group, the expressions of c-Fos were inhibited in inhibitors (D-AP5, NBQX) group. Data were presented as mean ± SEM. n=5/group. ** p < 0.01, *** p < 0.001.

To selectively activate the HPC-mPFC pathway, we used DREADD-based chemogenetic tools 38. Specifically, we microinjected the pAVV-CaMKIIα-hM3Dq-mCherry expressing excitatory DREADD hM3Dq into the HPC, and then 4 weeks later, microinjected CNO (1 mM, 0.5 µl/side) via the cannula implanted into the mPFC (Figure 3A-C). To verify whether CNO activates the HPC-mPFC pathway, we employed immunofluorescent double labeling of mCherry and c-Fos (a marker of neuronal activation), enabling us to visualize the expression of c-Fos and mCherry in the mPFC (Figure 3C-D). Compared with the control group, mice that received LPS exhibited a reduced number of c-Fos-positive neurons in the PFC, while the microinjection of CNO into the PFC of septic mice expressing hM3Dq increased c-Fos expression (Figure 3D: LPS+CNO vs. LPS, p < 0.001). Our results demonstrate that the HPC-mPFC pathway can be activated by a CNO injection.

### LPS-induced sepsis induces cognitive impairment and anxiety-like behaviors

**Figure 4:**
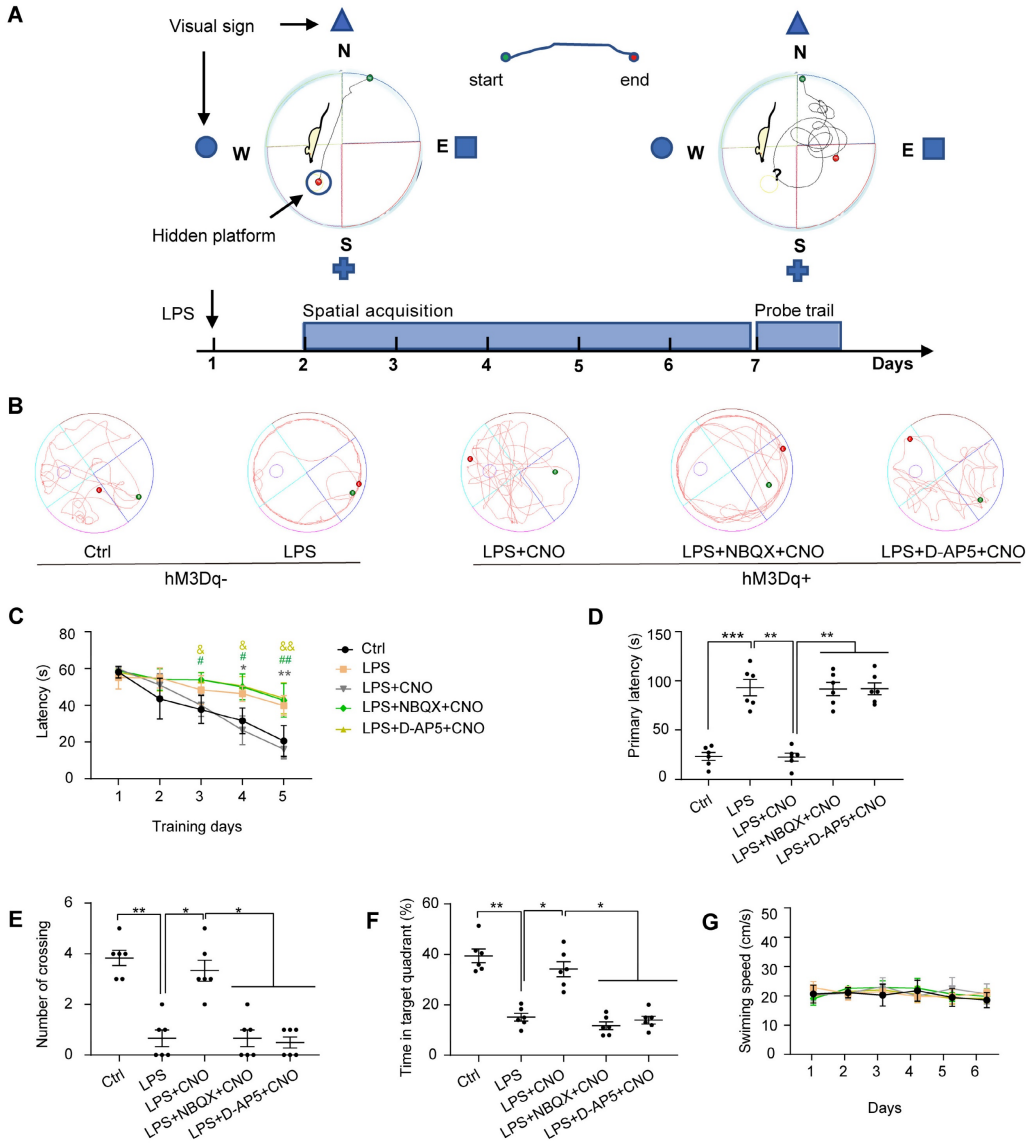
** The performance of the mice in the Morris water maze test.** (A) Experimental protocol of the Morris water maze (MWM). (B) Representative swimming paths during probe trial. (C) Activation of HPC-mPFC by CNO improves the LPS-induced learning deficits (*: CNO+LPS versus LPS; #: LPS+NBQX+CNO versus LPS; &: LPS+D-AP5+CNO versus LPS). (D-F) Activation of HPC-mPFC may ameliorate the LPS-induced spatial memory deficit, but the effect was blocked by glutamate receptor inhibitors (D-AP5 and NBQX). (G) Movement is unaffected. Data were presented as mean ± SEM. n=6/group. * p < 0.05, **: p < 0.01.

First, to test the effect of LPS-induced sepsis (LPS 5 mg/kg, i.p.) on cognitive function in mice, we performed a battery of behavioral tests associated with spatial learning and memory: the MWM, BM, and NOR. Representative swimming trajectories are shown in Figure 4B. In the spatial acquisition phase of the MWM, septic mice were much slower to learn to find the hidden platform compared with the Ctrl group (Figure 4C). During the memory test (probe trial) on day 6, in which the platform was removed, septic mice had a longer escape latency, fewer instances of crossing the area where the platform had been located, and spent less time in the target quadrant compared with mice in the Ctrl group (Figure 4D-F). We observed no differences in motor function in terms of swimming speed (Figure 4G).

**Figure 5:**
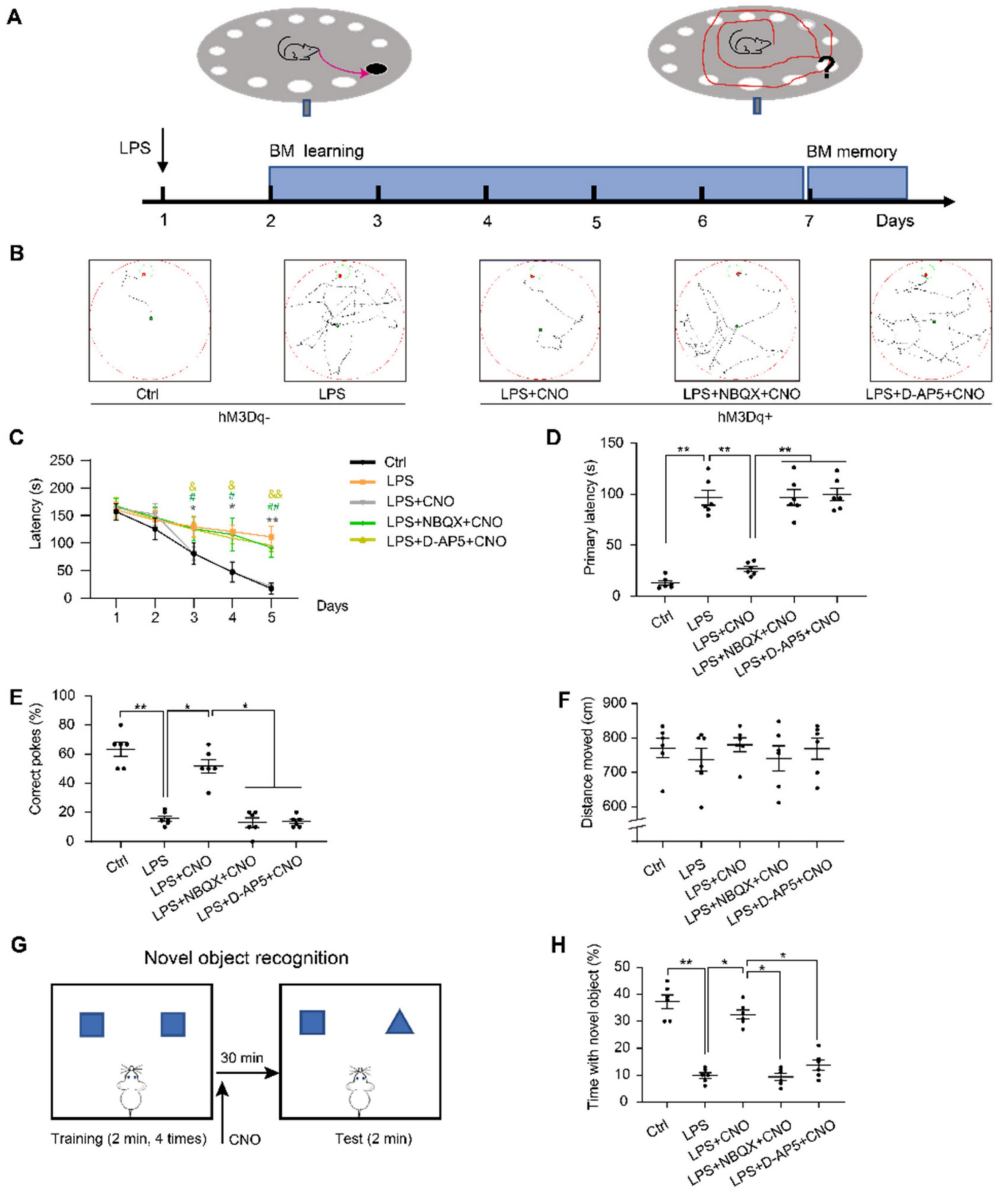
** The performance of the mice in the Barns maze and new object recognition test.** (A) Experimental protocol of the Barns maze (BM). (B) Representative searching paths during probe trial. (C) Chemogenetic activation of the HPC-mPFC pathway improves the LPS-induced learning deficits (*: CNO+LPS versus LPS; #: LPS+NBQX+CNO versus LPS; &: LPS+D-AP5+CNO versus LPS). (D-E) Activation of HPC-mPFC may improve the LPS-induced spatial memory deficit, but the effect was blocked by glutamate receptor inhibitors (D-AP5 and NBQX). (F) Motor function was not affected. (G) Experimental protocol for CNO injections using the new object recognition (NOR). (H) LPS group showed significantly worse recognition memory than controls, while activation of HPC-mPFC signaling pathway may improve the memory (LPS versus Ctrl; LPS+CNO versus LPS). This effect of HPC-mPFC was blocked by the glutamate receptor inhibitors (LPS+NBQX/D-AP5+CNO versus LPS+CNO). Data were presented as mean ± SEM. n=6/group. * p < 0.05, ** p < 0.01.

We then used the BM, which is considered to be less stressful than the MWM, to test spatial reference memory. As with the MWM, the LPS group spent longer exploring the target hole than the Ctrl group during the five consecutive learning days (Figure 5C). During the memory test on day 6, the LPS group had a longer primary latency and fewer correct pokes than the Ctrl group (Figure 5D, E). There were no significant differences in the distance moved between the two groups (Figure 5F). Figure 5B shows representative movement traces.

We administered the NOR (Figure 5G~H) to assess cognitive memory ability. We found that the septic mice explored the new object for less time than the mice in the Ctrl group (Figure 5H). These findings suggest that LPS-induced sepsis induces cognitive and spatial memory impairment in mice.

**Figure 6:**
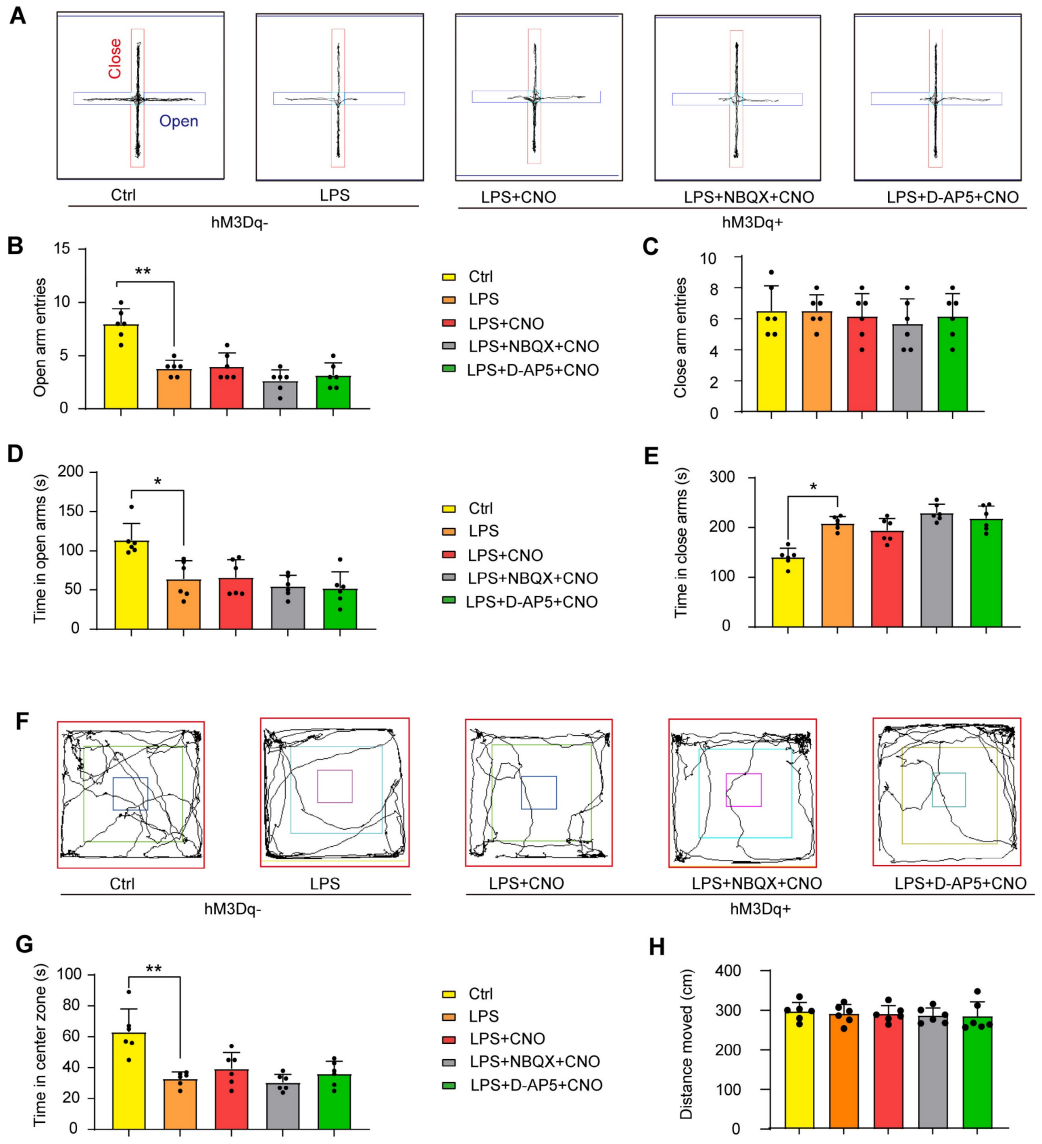
** Chemogenetic activation of the HPC-mPFC pathway do not improve anxiety-related behaviors in LPS-induced sepsis-associated encephalopathy.** (A-E) Activation of HPC-mPFC pathway did not alter LPS-induced anxiety-related behavior in the elevated plus maze (EPM) tests. (A) Representative searching paths on EPM. There were no significant differences in open arm entries (B), closed arm entries (C), time with open arm (D), and time with close arm (E) among LPS, Activator, and Inhibitors groups. (F-H) Activation of HPC-mPFC pathway did not improve LPS-induced anxiety-related behavior in the open field test (OFT). (F) Representative searching paths on OFT. (G) No significant difference was observed in time with center zone among LPS, Activator, and Inhibitors groups. (H) The distance moved in OFT (P > 0.05). Data were presented as mean ± SEM. n=6/group. * p < 0.05, ** p < 0.01.

We used the EPM and OFT to assess anxiety-like behaviors. Representative animal traces are shown in Figure 6A, F. In the EPM, the septic mice displayed fewer open-arm entries for both arms, and spent less time in the open arms compared with the mice in the Ctrl group (Figure 6B, D). Similarly, in the OFT, the septic mice spent less time than the control mice in the central area (Figure 6G). There was no obvious difference in the distance moved between the groups (Figure 6H). These results indicate that LPS-induced sepsis induces anxiety-like behaviors.

### Chemogenetic activation of the HPC-mPFC pathway improved LPS-induced cognitive dysfunction but not anxiety-like behavior

To investigate the role of the HPC-mPFC pathway in SAE, we first measured the effect of HPC-mPFC pathway activation on performance in the MWM, BM, and NOR. In the spatial acquisition phase of the MWM, the LPS+CNO group took less time to find the hidden platform than the LPS group (Figure 4C: * LPS+CNO vs. LPS). During the memory test (probe trial), the LPS+CNO group had a shorter escape latency, higher number of crossings of the region that had contained the platform, and spent more time in the target quadrant compared with the LPS group (Figure 4D-F). There were no significant differences in exercise ability in terms of swimming speed between the groups (Figure 4G). Representative swimming traces are shown in Figure 4B. Similarly, during the learning phase in the BM test, mice in the LPS+CNO group spent less time than the septic mice exploring the target hole (Figure 5C: *LPS+CNO vs. LPS). During the BM memory test, mice in the LPS+CNO group also had a shorter primary latency and more correct pokes than the LPS group (Figure 5D, E). The distance moved was not significantly different between the groups (Figure 5F). Figure 5B shows representative movement traces. In the NOR task (Figure 5G~H), the mice in the LPS+CNO group spent more time exploring the new object compared with the LPS group (Figure 5H). These behavioral data indicate that chemogenetic activation of the HPC-mPFC pathway improved LPS-induced cognitive dysfunction.

We also measured the effects of HPC-mPFC pathway activation on performance in the EPM and OFT. However, we found no significant differences in behavioral performance between the LPS+CNO and LPS group (Figure 6).

### Inhibition of glutamate receptors abolished the effects of HPC-mPFC activation and blocked activation of the HPC-mPFC pathway

To learn how glutamate receptors and associated downstream signaling pathways interact with the HPC-mPFC pathway, we assessed behavioral performance and c-Fos expression via microinjection of a glutamate receptor inhibitor 30 min before a CNO injection in the mPFC. In terms of the MWM, BM, and NOR tests, the mice in all of the inhibitor groups performed significantly worse than those in the Activator group on tests of spatial memory and cognitive function (Figure 4, Figure 5: LPS+NBQX/D-AP5+CNO vs. LPS+CNO). These data indicate that inhibitors of glutamate receptors abolished the effects of HPC-mPFC activation and blocked the activation of the HPC-mPFC pathway.

### The glutamate receptor-mediated CaMKII/CREB/BDNF/TrKB signaling pathway influenced the role of the HPC-mPFC pathway in sepsis-induced cognitive dysfunction

**Figure 7:**
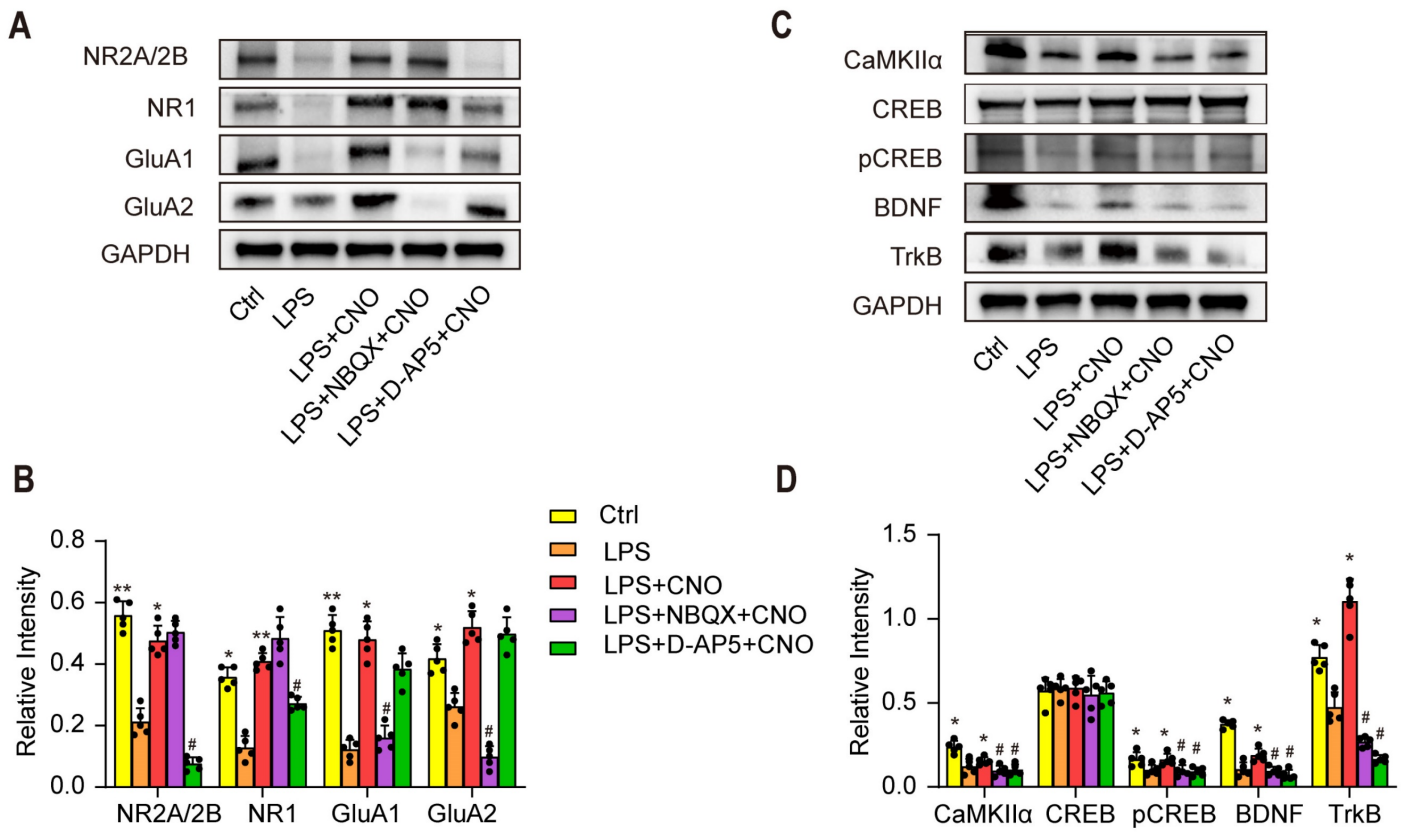
** The glutamate receptor-mediated CaMKII/CREB/BDNF/TrKB signaling pathway influenced the role of the HPC-mPFC pathway in sepsis-induced cognitive dysfunction.** (A-B) LPS reduces levels of glutamate receptors (AMPAR subunit GluA1/2, NMDAR subunit NR2A/2B and NR1) in mPFC, while activation of HPC-mPFC abolishes the decrease. AMDAR inhibitor (NBQX) and NMDAR inhibitor (D-AP5) effectively reduce the express of AMDAR (GluA1/2) and DMDAR (NR2A/2B and NR1) (* LPS+CNO versus LPS, ^#^ LPS+NBQX/D-AP5+CNO versus LPS+CNO). (C-D) NBQX and D-AP5 effectively reduce the express of CaMKIIα, pCREB/CREB, BDNF, and TrkB in mPFC, while activation of HPC-mPFC prevents the decrease (* LPS+CNO versus LPS, ^#^ LPS+NBQX/D-AP5+CNO versus LPS+CNO). Data were presented as mean ± SEM. n=5/group. * p < 0.05, ** p < 0.01, ^#^ p<0.05, ^##^ p<0.01.

To further explore the molecular mechanisms by which the HPC-mPFC pathway regulates cognitive dysfunction in sepsis-induced brain injury, we used WB to examine the expression levels of AMPAR (GluA1, GluA2), NMDAR (NR1, NR2A/2B), and downstream signaling molecules including CaMKII, pCREB/CREB, BDNF, and TrKB in the mPFC (Figure 7A~D). We found that the levels of AMPAR (GluA1, GluA2), NMDAR (NR1, NR2A/2B), CaMKII, pCREB/CREB, BDNF, and TrKB increased significantly after activation of the HPC-mPFC pathway (Figure 7 A~D; * p < 0.05, ** p < 0.01), and that obstructing glutamate receptors via NBQX (AMPAR inhibitor) or D-AP5 (NMDAR inhibitor) abolished this increase (Figure 7A~B, C~D: ^#^ P < 0.05, ^##^ P < 0.01). These data suggest that the glutamate receptor-mediated CaMKII/CREB/BDNF/TrKB signaling pathway influenced the role of the HPC-mPFC pathway in sepsis-induced cognitive dysfunction.

## Discussion

Sepsis has a high mortality rate, and thus remains a major public health problem worldwide. How, the mechanisms by which sepsis affects cognitive function are unclear. DREADD-based chemogenetic technology has become increasingly popular in the field of neuroscience 38. It can be used to identify neurocircuits associated with behavior, cognition, and emotions in animals ranging from flies to nonhuman primates 38. Here, we performed a projection-specific manipulation to examine the role of the HPC-mPFC pathway in cognitive dysfunction in septic mice. Our study has shown that activation of the HPC-mPFC pathway improved cognitive function in normal mice. This result is consistent with the previous reports revealing that HPC-PFC input promote spatial memory 3, 15. Moreover, we also demonstrated that chemogenetic activation of the HPC-mPFC pathway improved cognitive function in septic mice. This suggests that abnormal function of the HPC-mPFC pathway is implicated in the pathogenesis of sepsis-induced brain injury. These observations indicated that the HPC-mPFC pathway played an important role in cognitive dysfunction induced by sepsis. Alternatively, the glutamate receptor inhibitors did not alter behavior in both control mice (non-LPS) as well as in LPS treated mice expressing pAAV-CaMKIIα-mCherry. This might be partly explained by that glutamate receptor inhibitors had no effect on resting potential in normal mice 39. Separately, as shown by other studies, we also found that AMPAR and DMDAR were down-regulated significantly after LPS injection 40. This could probably explain why glutamate receptor inhibitors did not alter behavior in LPS treated mice expressing pAAV-CaMKIIα-mCherry. Although there still were some studies mentioned that injection of D-AP5 in the hippocampus might affect partially cognitive function, these injection sites were done directly in the hippocampus 41, 42. Few studies have reported the role of D-AP5 or NBQX injection in mPFC on cognitive function. Furthermore, our studies show that the effect of HPC-mPFC activity in improving cognitive function in septic mice could be blocked by glutamate receptor (AMPAR or DMDAR) inhibitors. This suggested that the effects of HPC-mPFC signaling pathway may be regulated by glutamate receptor. Impairment of the HPC-mPFC pathway via LPS reduced levels of postsynaptic glutamate receptors (AMPAR and NMPAR), inhibited activity in the CaMKII/CREB/BDNF/TrKB signaling pathway, and diminished spatial cognitive function in mice (Figure 7). Therefore, the glutamate receptor-mediated CaMKII/CREB/BDNF/TrKB signaling pathway appears to modulate the role of the HPC-mPFC pathway in LPS-induced cognitive dysfunction. Our data reveal a novel mechanism by which the HPC-mPFC pathway is involved in sepsis-related cognitive dysfunction, thus providing new evidence for therapeutic targets for SAE.

C-fos is a transcription factors typically expressed in the nucleus 43. However, our present study shows that the c-fos staining in Figure 3 is largely cytoplasmic. The c-Fos level is very low without stimulation and induced rapidly at the transcriptional level by a number of stimuli. Research has shown that c-fos proteins left the nucleus for the cytoplasm when proliferation was stopped by the high density of culture cells or by serum starvation in cultures 44. The fact that the cFos protein would be present in cytoplasm for at least a few hours after the onset of activation has made identification of the stimulated neurons possible 43. This led us to speculate that c-fos could rapidly translocate from nuclear to cytoplasm after CNO-induced activation. This point needs further experimental confirmation.

Both the LPS model and cecal ligation and puncture (CLP) model of SAE have been used to create short- and long-term behavioral impairments, including changes in non-aversive (MWM, OFT, EPM, NOR) and aversive memory (fear conditioning) 45. Compared with the CLP model, cognitive dysfunction can be assessed earlier after the initiation of LPS-induced SAE because movement is less limited after the procedure 45. In the present study, we comprehensively examined the early impact of LPS-induced sepsis on cognitive and anxiety-related behavioral parameters in mice. We found that LPS-induced sepsis not only impaired cognitive function, but also altered affective behaviors. These behavioral results are partially consistent with previous reports 32, 46, 47. However, one previous study did not find evidence of both impaired memory and affective behavior in the MWM, NOR, and OFT one month following the injection of LPS (5 mg/kg) 36, and cognitive dysfunction was intact 30 days after the administration of LPS (5 mg/kg) for 7 consecutive days in one study 48. In addition to the LPS dosage and mouse strain, factors including mouse sex and age, the length of the interval between the model induction and test, the timing of the training and test, etc., may influence both the degree of cognitive impairment and the results of behavioral tests 45.

Many therapeutic approaches have been used to prevent sepsis-induced cognitive impairment in mice. These include acetylcholinesterase inhibitors 49, erythropoietin 32, ACE inhibitors 50, morphine 51, the benzodiazepine agonist MRK-016^52^, the NMDA antagonist MK-801 53, and others. Among these strategies, anti-oxidative and anti-inflammatory treatments were the most frequently used, which demonstrates the importance of oxidative stress and inflammatory pathways on the pathogenesis of SAE. Non-pharmacological methods including physical exercise 54 and environmental rehabilitation 55 have also been used to address cognitive impairment after sepsis. However, the neurological pathways associated with SAE have not been previously reported. HPC projections to the mPFC are thought to be associated with memory 3, 56 and anxiety regulation 57. Further, the HPC-mPFC pathway is important for encoding task-relevant location cues 3. Here, we verified that the vHPC projects to the mPFC at the structural level in C57BL/6 mice (Figure 2). Then, we observed the function of this projection in terms of alterations in memory and anxiety-associated behaviors. By selectively regulating the mPFC-vHPC projection via chemogenetics, we found that the HPC-mPFC pathway regulates spatial memory, but not anxiety-associated behaviors in SAE mice. This has not been previously reported, and thus needs to be confirmed by further studies.

Ionotropic glutamate receptors (iGluR) are the main mediators of excitatory synaptic transmission in the brain 58. NMDAR-dependent plasticity in the HPC is vital for episodic-like memory. Further, the activation of NMDA receptors is key for memory encoding, and can trigger memory formation 59. The results showed that the expressions of glutamatergic receptor were down-regulated significantly after LPS injection. This is consistent with previous results reported by Wu et al 40, 60. In their study, they found that a significant decrease in GluR1 and NMDA receptor (NR1) after four consecutive days of LPS injection. Regarding glutamatergic receptors, previous reports have indicated that alterations of NMDAR subunits mediate synaptic plasticity impairment in the prefrontal cortex (PFC) and hippocampus (HPC) of LPS-induced septic mice 61, 62. NMDARs, comprising NR1, NR2A and 2B, and NR3 subunits, have different features and functions 63. Regarding neuronal development and memory consolidation in mice, some studies have revealed that the NR2A/2B subunits of NMDA mediate synaptic plasticity and learning ability 64, 65. Jonatan et al 66 have demonstrated that downregulation of NR2A/2B subunits is associated with cognitive impairment in a pristane-induced lupus BALB/c mice. Our study showed that pharmacological blockade of NMDA receptors reversed the effects of activation mainly on NR2A/B expression while AMPA receptor antagonists block the effects on AMPAR subunit GluA1/2 expression. This suggests that the NR2A/B and GluA1/2 subunits play a crucial role in neuroinflammation-related cognitive impairment.

AMPARs enable information transfer via NMDAR-dependent synaptic plasticity, specifically, by alleviating voltage-dependent channel blocking through Mg^2+^, allowing postsynaptic Ca^2+^ entry, and enhancing excitatory synaptic transmission 67. In some synapses, AMPARs can also directly mediate Ca^2+^ influx, thus enabling the regulation of postsynaptic plasticity 67. A Ca^2+^ influx can contribute to the activation of CaMKII, which induces long term potentiation and enhances learning and memory 68. CaMKII phosphorylates the transcription factor CREB, and converts it into active form p-CREB 69. CREB activation results in BDNF transcription in the hippocampus 70. As an important molecule involved in memory, BDNF binds to the TrkB receptor, thus promoting excitatory signaling and long-term potentiation 71. In the present study, we detected the expression of both AMPAR and NMDAR, and their downstream signaling molecules, including CaMKII, CREB, pCREB, BDNF, and TrKB in the mPFC. Our data showed that the effect of HPC-mPFC in improving cognitive function could be blocked by both glutamate receptor (AMPAR or DMDAR) inhibitors. The levels of glutamate receptors (AMPAR or DMDAR) and CaMKII/CREB/BDNF/TrKB were reduced by LPS, but increased by the activation of the HPC-mPFC pathway. The increased expression of both AMPAR and NMDAR activated the downstream CaMKII/CREB/BDNF/TrKB signaling pathway after targeted HPC-mPFC activation. These results indicate that the glutamate receptor modulates the role of the HPC-mPFC pathway in sepsis-induced cognitive dysfunction.

This study represents an innovative exploration in the field of SAE research, as previous studies had not paid sufficient attention to the relationship between the HPC-PFC pathway and SAE. Therefore, this experiment fills a critical gap in our understanding. Given the high mortality rates associated with sepsis and SAE being one of its most common complications, severely affecting both survival outcomes and quality of life for septic patients, this experiment emphasizes clinical needs and cutting-edge global issues. By adopting various techniques such as behavioral studies, chemical genetics, anterograde and retrograde tracing, etc., this study provides the first evidence from a neural projection pathway level that HPC-PFC pathway plays a vital role in mediating cognitive impairment in SAE. As a result, it proposes new insights into the pathogenesis of SAE and potential therapeutic targets that are of great significance and innovation.

Our study has some limitations. First, CNO can be reverse metabolized to clozapine which itself is biologically active and can produce off-target effects. CNO has very good drug-like properties with rapid CNS diffusion and distribution, and has at least a 60 min residence *in vivo* in mice 38. To avoid the off-target effects, on the one hand, intracranial injection was used to prolong residence time of CNO in brain; on the other hand, time of behavioral test per mouse was controlled within half an hour as promptly as possible. Second, some studies revealed sex differences in acute immune response and neuro-inflammatory response after exposure to LPS and possible mechanisms involved in the enduring alterations in behavior and brain function following LPS treatment 72-74. Given that all studies were conducted in male mice, our findings may not be generalizable. Besides, the effect of inhibiting the HPC-mPFC on learning and memory by chemogenetic approach has not been tested. Thus, this study can merely demonstrate the role of glutamate receptors in modulating spatial memory deficits induced by sepsis via HPC-mPFC pathway, without specifying its causal link. It is crucial to conduct more in-depth studies, including inhibitor experiments, in the future to further investigate the specific mechanisms and causal relationships of HPC-mPFC pathway in cognitive impairment caused by sepsis.

## Conclusions

In summary, we identified a prominent excitatory connection between the HPC and mPFC, and revealed the vital role of the HPC-mPFC pathway in improving cognitive dysfunction after LPS-induced brain injury. Further, the glutamate receptor appears to be an important molecular mechanism linking the HPC-mPFC pathway with cognitive dysfunction in SAE.

## Abbreviations

SAE: sepsis-associated encephalopathy
CNS: central nervous system
LPS: lipopolysaccharide
HPC: hippocampus
vHPC: ventral hippocampus
PFC: prefrontal cortex
mPFC: medial prefrontal cortex
CNO: clozapine-N-oxide
DREADD: designer receptors exclusively activated by designer drugs
GPCR: G protein coupled receptors
GRS: glutamate receptor signaling pathway
AMPAR: alpha-amino-3-hydroxy-5-methyl-4-isoxazolepropionic acid receptor
NMDAR: N-methyl-D-aspartate receptor
MWM: Morris water maze
BM: Barnes maze
NOR: new object recognition
OFT: open field test
EPM: elevated plus maze
CaMKII: calmodulin-dependent kinase II
CREB: cAMP-response element binding protein
BDNF: brain-derived neurotrophic factor
TrKB: tyrosine receptor kinase B
AAV: adeno-associated virus
